# Exploring infant feeding practices: cross-sectional surveys of South Western Sydney, Singapore, and Ho Chi Minh City

**DOI:** 10.1186/s12887-017-0902-0

**Published:** 2017-06-13

**Authors:** Timothy Yong Qun Leow, Andrew Ung, Shelley Qian, Jessie Thanh Nguyen, Yvonne An, Poonam Mudgil, John Whitehall

**Affiliations:** Department of Paediatrics, School of Medicine, Western Sydney University, Sydney, NSW Australia

**Keywords:** Infant feeding, Breast feeding, Paediatric nutrition, Obesity, Australia, Vietnam, Singapore

## Abstract

**Background:**

Infant feeding practices are known to influence the child’s long-term health. Studies have associated obesity and other diseases with reduced breastfeeding and early introduction of high calorie beverages (HCBs). The rising prevalence of obesity is already a problem in most developed countries, especially Australia, but cultural differences are influential. Our aim is to examine and compare infant feeding practices and educational levels of respondents through questionnaires in three culturally different sites: Campbelltown (South Western Sydney), Australia, Singapore and Ho Chi Minh City, Vietnam (HCMC).

**Methods:**

Consenting parents and carers (aged ≥18 years old) of at least one child (≤6 years old) were recruited from paediatric clinics in Campbelltown, Singapore and HCMC. Participants completed an infant feeding practices questionnaire regarding breastfeeding, beverage and solid initiation in addition to the parent’s ethnicity, age, and educational level. Data was analysed quantitatively using SPSS.

**Results:**

Two hundred eighty-three participants were recruited across the three sites, HCMC (*n* = 84), Campbelltown (*n* = 108), and Singapore (*n* = 91). 237 (82.6%) children were breastfed but in all only 100 (60.2%) were exclusively breastfed for five months or more. There was a statistical difference in rates of breast feeding between each region. HCMC (*n* = 18, 21.4%) had the lowest, followed by Campbelltown (*n* = 35, 32.4%), and then Singapore (*n* = 47, 51.7%). There was also a difference in rates of introduction of HCBs by 3 years of age, with those in HCMC (*n* = 71, 84.5%) were higher than Campbelltown (*n* = 71, 65.8%) and Singapore (*n* = 48, 52.8%). The educational level of respondents was lower in Vietnam where only 46.4% (*n* = 39) had completed post-secondary education, compared to 75.0% (*n* = 81) in Campbelltown and 75.8% (*n* = 69) in Singapore.

**Conclusions:**

Rates of breast feeding were inversely correlated with rates of introduction of HCB and positively related to educational achievement. Vietnam had lowest rates of breast feeding, higher rates of introduction of HCBs, and lower rates of education. Given rising rates of obesity, there is a need for more effective programmes to promote breast feeding and restrict false advertising of HCBs.

**Electronic supplementary material:**

The online version of this article (doi:10.1186/s12887-017-0902-0) contains supplementary material, which is available to authorized users.

## Background

Appropriate nutrition is fundamental for a child’s development. Inappropriate feeding practices, such as a shorter length of breastfeeding and early introduction of solids or high calorie beverages (HCBs), have been associated with dental caries [1], diabetes [2], nutritional deficits and bone catabolism [3], hypertension [4], obesity [5–7], and metabolic syndrome as adults [8].

WHO guidelines [9] recommend exclusive breastfeeding for the first 6 months, introduction of complementary foods at 6 months (but not before 4 months), and concurrent breastfeeding until 24 months or longer [10–12]. Australia, Vietnam and Singapore have similar guidelines [13, 14].

Breast milk contains all the required nutrients for growth in the first six months of life [15]. It has been associated with better cognitive development, and the prevention of such negative health outcomes as infections (gastrointestinal and respiratory), obesity, diabetes, cardiovascular disease, and sudden infant death [10, 15–17].

Formula-fed infants gain weight more quickly and have higher BMI at 6 years of age than their breastfed counterparts [18–20]. Reasons include the higher protein content of infant formula and parental desire to ensure the whole bottle is emptied each feed [18]. A faster growth rate is, however, associated with a higher incidence of obesity [20] and, therefore, a delay in solid food introduction until 24 weeks appears to reduce risk of child obesity at 10 years [6].

HCBs contribute to obesity [5, 21, 22]. They include soft drinks, energy drinks and fruit drinks with added sugar [23–25]. Earlier introduction not only provides excess calories in infancy but is known to induce sustained consumption in later years [26], predisposing to obesity and metabolic syndrome [5, 7, 27]. The addition of caffeine to HCBs compounds the problems of obesity with predisposition to hyperactivity and addictive behaviour in children [21, 28–30].

Childhood obesity affects psychological and physical health, including orthopaedic and respiratory problems and the advent of metabolic syndrome [31–33]. In Singapore, in 2012, the rate of clinical childhood obesity was reported to be 11%, but is now increasing [34, 35]. In 2011–12, some 25% of Australian children aged 5–17 years were reported to be overweight [36], with 7.6% being clinically obese [37]. In Vietnam, in 2007–13, the rate of clinical obesity in children <5 years old was reported to be between 11.5–16.3% [38, 39]. Rates of obesity are generally twice as high in countries of greater income [40–42].

As cultural practices influence feeding, we aimed to investigate and compare infant feeding practices at three sites with cultural and demographic differences. Campbelltown in South-Western Sydney (SWS), Singapore and Ho Chi Minh City (HCMC) [43, 44]. SWS, a lower socioeconomic region of Sydney has a culturally diverse population [45] of which about 40% come from non-English speaking backgrounds. Its birth rate is higher than the Australian average. Singapore [46] is an established “middle-class” state, with an ageing population, predominately Chinese ethnicity and four official languages, English, Mandarin, Tamil, and Malay. HCMC is the largest city in Vietnam [47] a developing economy whose majority is of the Viet (Khin) ethnicity and language.

## Methods

### Data collection

A voluntary, self-reported survey was administered to parents or carers, eighteen years and over, who were responsible for children less than six years old. The participants were approached randomly at the three sites: outpatient clinics and public parks in the vicinity of Campbelltown Hospital, Xom Moi medical centre in HCMC, and Singapore General Hospital. Data collection occurred between December 2014 and February 2015.

Participants were given a participant information sheet and an explanation of the project before the survey was completed. Participants were excluded if they were not fluent in English or Vietnamese; or were illiterate.

### The various drinks were defined as below

Cordial: flavoured syrup that may be mixed with water.

100% fruit juice: either self-juiced from fresh fruit or bottled and claimed by manufacturer to contain 100% natural fruit without additives.

Fruit drink: juice without associated claims to be totally comprised of fruit derivatives.

Non-caffeinated soft-drinks: sweetened carbonated beverages, without caffeine.

Caffeinated soft-drinks: sweetened carbonated beverages, advertised to contain caffeine with an average concentration of 30 mg per 375 ml.

Energy drinks: soft-drinks with ≥80 mg of caffeine per 375 ml (1 can).

High caloric beverages (HCBs): cordial, flavoured milk, 100% fruit juice, fruit drink, non-caffeinated and caffeinated soft-drinks, and energy drinks, all containing around 10% glucose.

Caffeinated drinks (CDs): caffeinated soft-drinks, energy drinks, coffee, and tea.

### The infant feeding questionnaire

The survey consisted of basic demographic questions of the parent or carer including gender, age, level of education, preferred language, place of birth and number of children being cared for. Questions relating to the youngest child’s diet included the age to which the child was exclusively breastfed, whether any of the listed drinks had been commenced in the child’s diet, at what age they were introduced and the frequency of consumption. Questions also included the age of introduction of solid foods, the types of those solid foods and their frequency of consumption (See Additional file 1).

The questionnaire was conducted in English in Australia and Singapore, and Vietnamese in Vietnam. The three cities were selected in part due to convenience sampling. There are also unique demographic qualities which we wanted to compare, culturally – in relation to the infant feeding practices [Ho Chi Minh – a predominantly Eastern culture city in a developing country; Singapore – a mixed Eastern-Western culture in an ex-British colony in a developed country; South-Western Sydney – a predominantly Western culture region in a developed country].

All completed questionnaires were kept confidential and anonymous. This project was approved from the University of Western Sydney Human Research Ethics Committee (HREC H9140, H9067), Liverpool Local Health District for the site at Campbelltown Hospital (HREC/13/LPOOL/153 and SSA/13/LPOOL/154), Singapore General Hospital (CIRB 2015/2078), and Xom Moi Medical Centre certification.

### Statistical analysis

The data from the survey were entered into SPSS 20 and all statistical analyses were conducted using SPSS. Descriptive statistics of the demographic features for the participants and the youngest child were analysed. Line graphs showing the cumulative frequencies by age for the introduction of different drinks by location were produced. A one-way Analysis of Variance (ANOVA) was performed between location and age of introduction of the various drinks, and location and age of exclusive breastfeeding. Each drink was individually analysed using ANOVA consisting of one independent variable (with three levels: HCMC, Campbelltown, and Singapore) and one dependent variable (the age of introduction of that drink). Post-hoc Tukey HSD analysis was performed on statistically significant (*p* < 0.05) results from the ANOVA test.

## Results

Two hundred eighty-three participants completed the written questionnaire: 108 from Campbelltown, 91 from Singapore, and 84 from HCMC. Of the 283 respondents, 37 (12.9%) were male and 246 (87.5%) were female. The ages of the respondents ranged from 20 to 70 with the majority between 25 to 39 (79.8%, 226/283). Most respondents had completed tertiary education (59.2%, 170/283). 77.7% (220/283) of participants cared for 2 or less children (Additional file 2).

The children in the three locations were similar: The median age of the child which was the subject of the questionnaire was 19 months (IQR 25) and gender rates were very similar between sites: 150 (53.0%) were boys and 133 (47.0%) girls (Additional file 3).

**Fig. 1:**
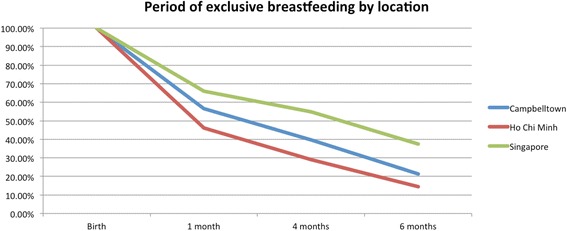
Percentage of participants that were still exclusively breastfeeding by location

Overall, 64.4% of 283 participants began to feed their children exclusively with breast milk, but this number dropped to 41.2% at four months, and 24.4% at six months (Fig. 1). In HCMC, 59.5% of mothers began exclusively breastfeeding but this dropped to 29.0% at four months, and 14.5% at six months. In Campbelltown, 61.1% of mothers began exclusively breastfeeding but this dropped to 39.8% at four months and 21.3% at six months. In Singapore, 67.0% of mothers began exclusively breastfeeding but this rate dropped to 55.0% at four months and 37.4% at six months. The duration of exclusive breastfeeding differed significantly between locations, *F*(2189) = 29.29, *p* < 0.005. A Post-hoc Tukey test showed it was significantly lower in Vietnam than Campbelltown (*p* < 0.005), and in Vietnam compared to Singapore (*p* < 0.005). There was no significant difference between Campbelltown and Singapore.

**Fig. 2:**
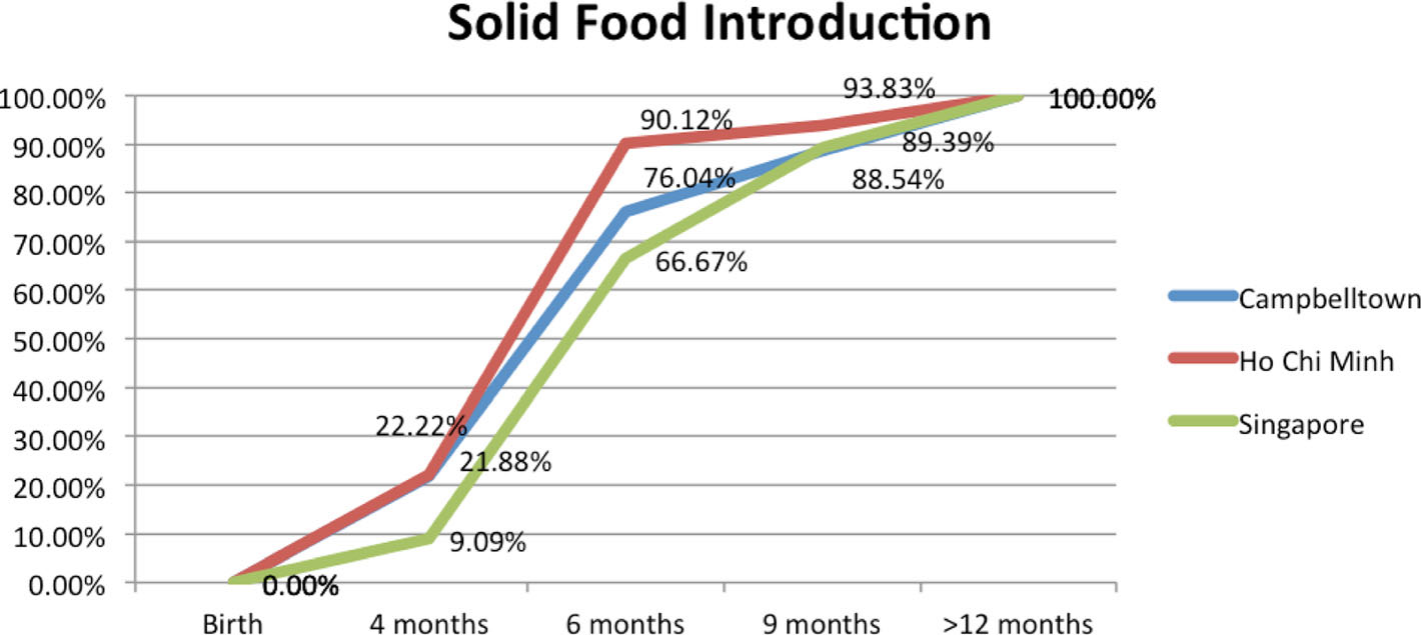
Age of solid food introduction by age across locations

The percentage of children being introduced solid foods at four months was 21.9% in Campbelltown, 22.2% in HCMC, and 9.1% in Singapore. By six months the percentage of children receiving solid foods was 76.0% in Campbelltown, 90.1% in HCMC, and 66.7% in Singapore (Fig. 2). These rates were not statistically different.

**Fig. 3:**
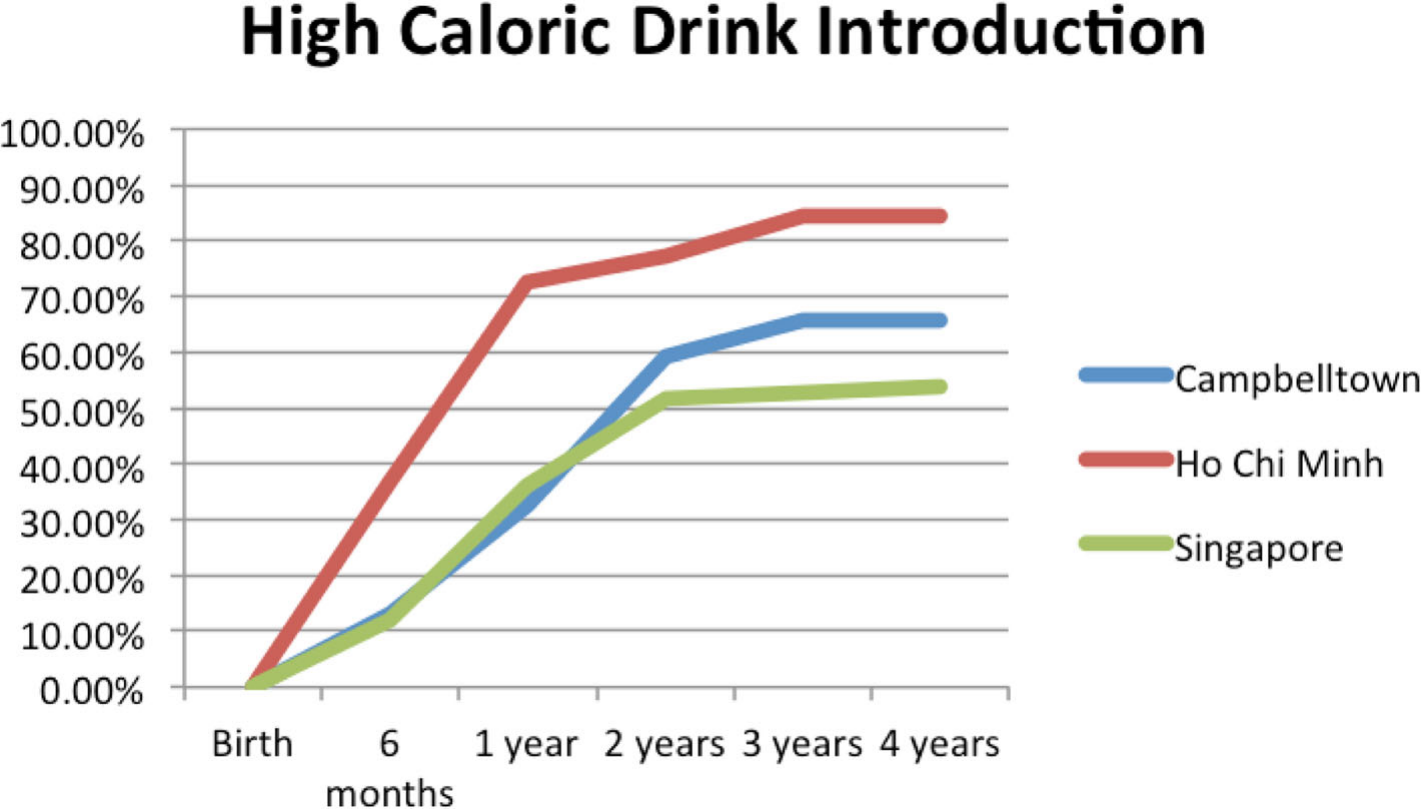
Number of participants who introduced any high calorie beverage by age across locations

**Fig. 4:**
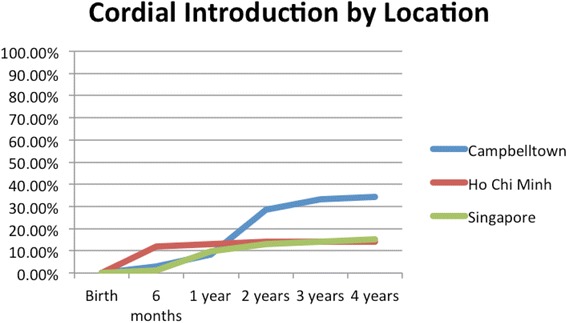
Number of participants who introduced cordial by age across locations

**Fig. 5:**
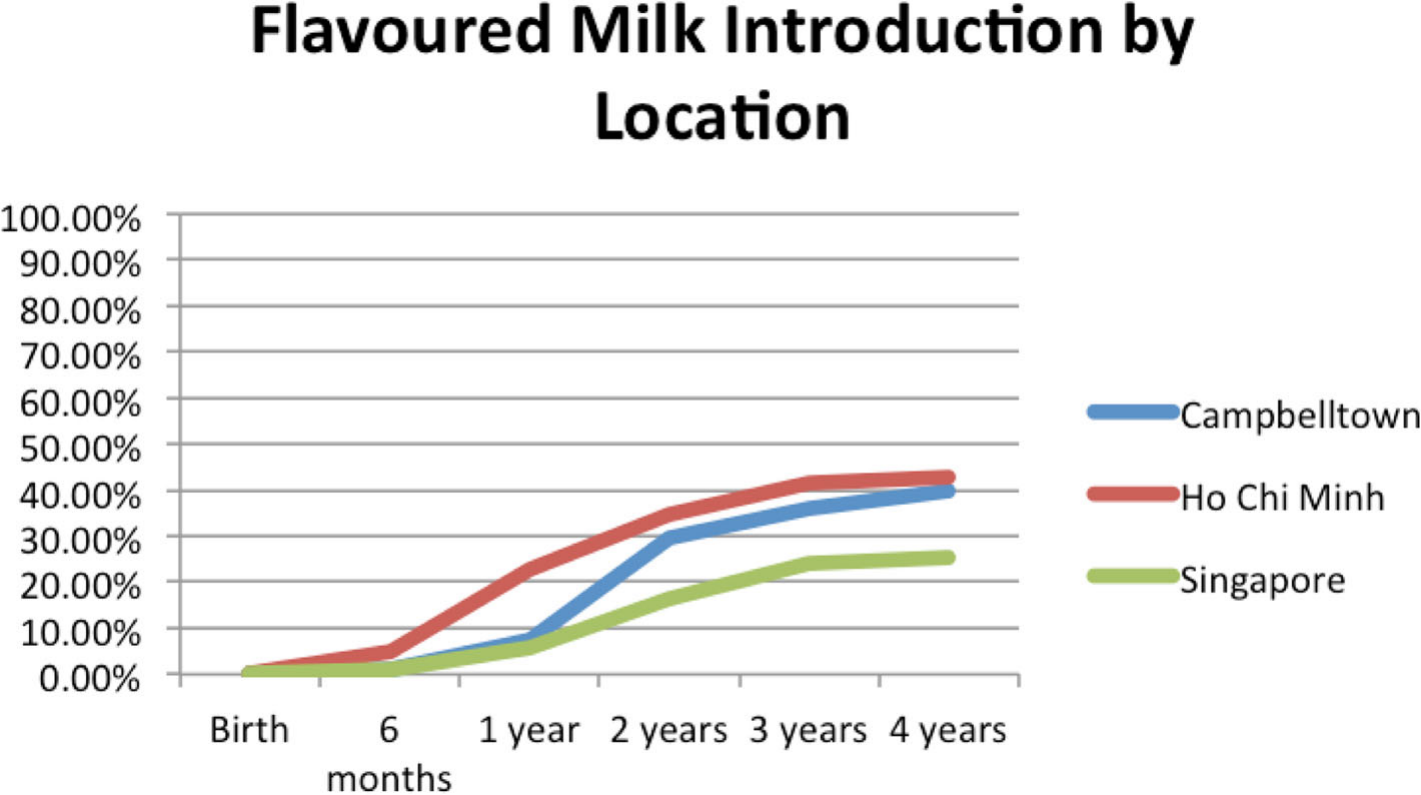
Number of participants who introduced flavoured milk by age across locations

**Fig. 6:**
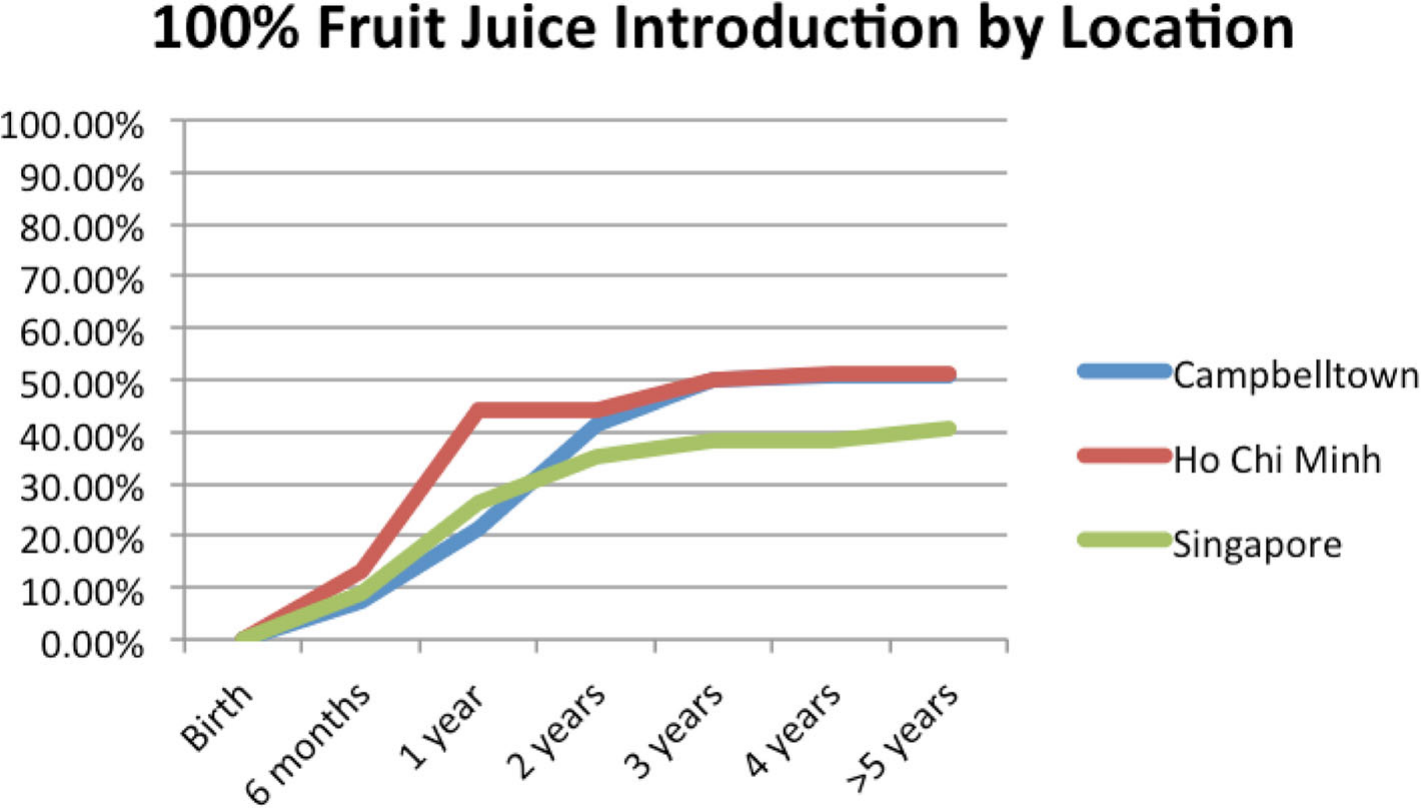
Number of participants who introduced 100% fruit juice by age across locations

**Fig. 7:**
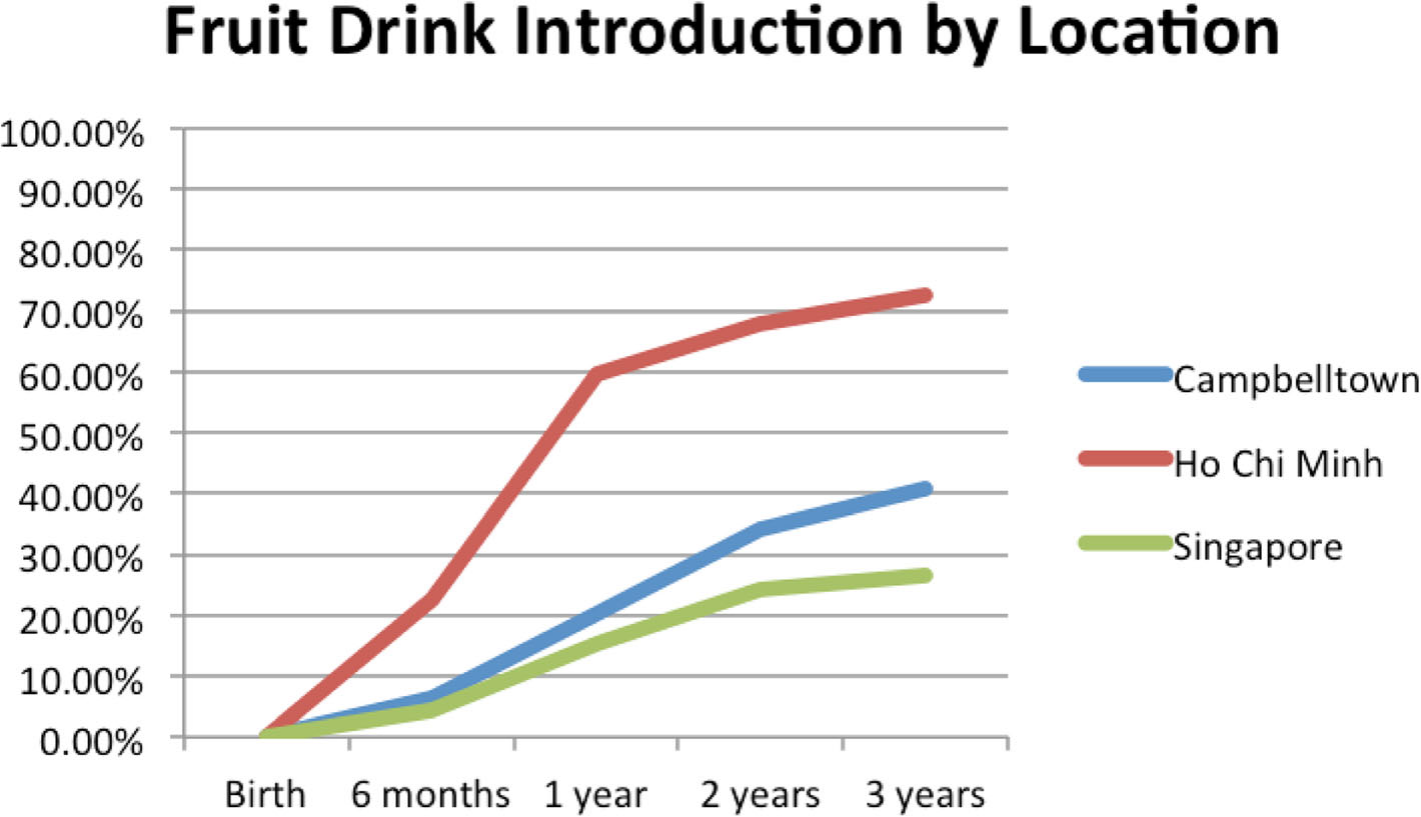
Number of participants who introduced fruit drink by age across locations

**Fig. 8:**
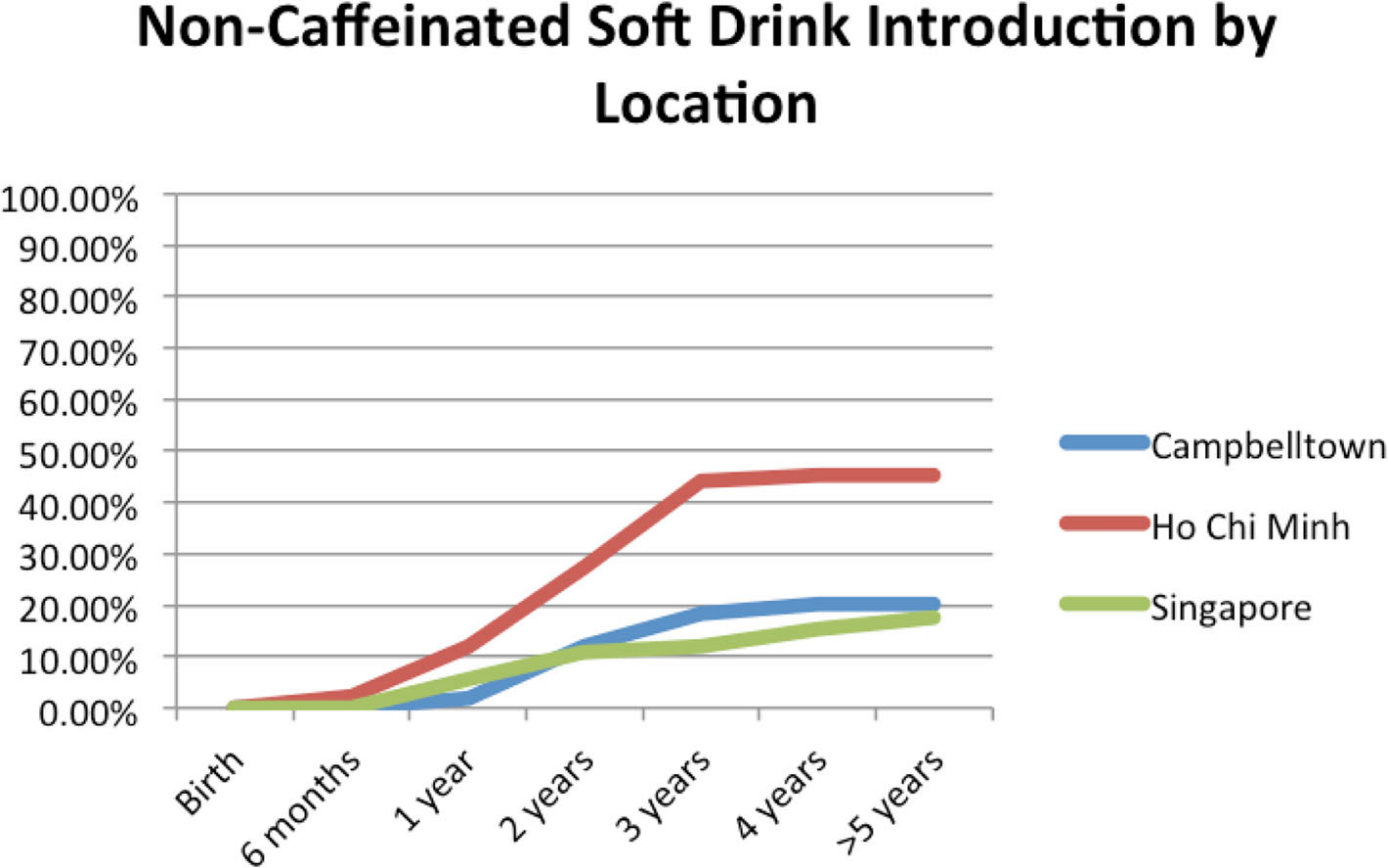
Number of participants who introduced non-caffeinated soft drink by age across locations

**Fig. 9:**
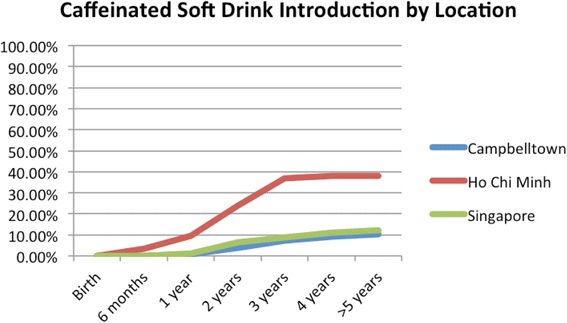
Number of participants who introduced caffeinated soft drink by age across locations

**Fig. 10:**
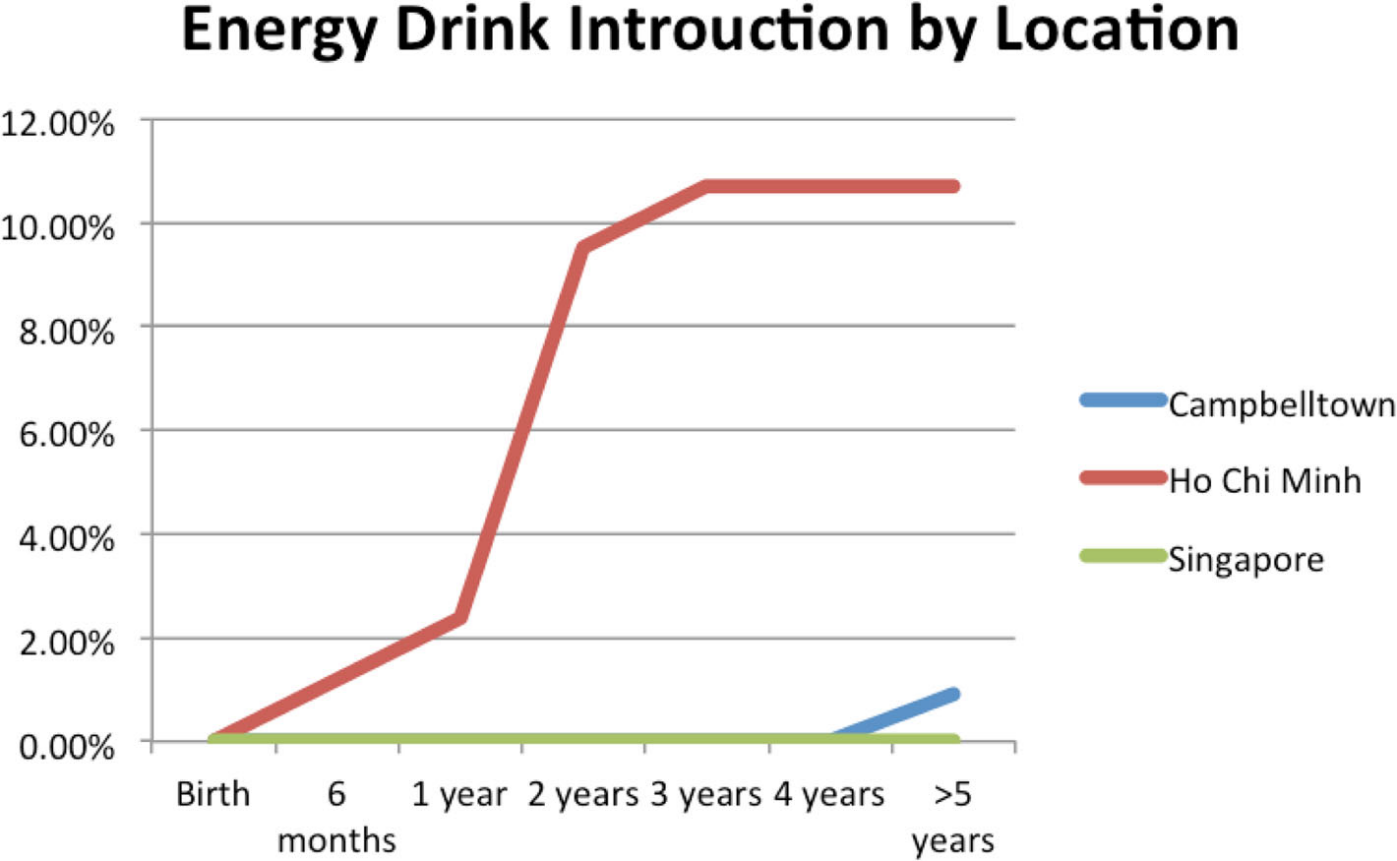
Number of participants who introduced energy drinks by age across locations

HCBs, such as cordial, flavoured milk, 100% fruit juice, fruit drink, and non-caffeinated soft drinks (Figs. 3, 4, 5, 6, 7, 8, 9 and 10), were introduced at an early age in all localities, but more frequently in Vietnam. 36.9% of respondents in HCMC reported giving HCBs to children at six months or less, compared with 13.0% in Campbelltown and 12.1% in Singapore. At one year, 72.6% of participants in HCMC had introduced HCBs, compared to 32.4% in Campbelltown and 36.3% in Singapore. By three years of age, 84.5% of participants in HCMC had introduced HCBs, compared to 65.8% in Campbelltown and 52.8% in Singapore. These differences were statistically significant for both age and rate of introductions *p* < 0.05. HCMC had introduced more HCBs and at an earlier age than the other sites at a statistically significant rate to children (≤3 years old).

**Fig. 11:**
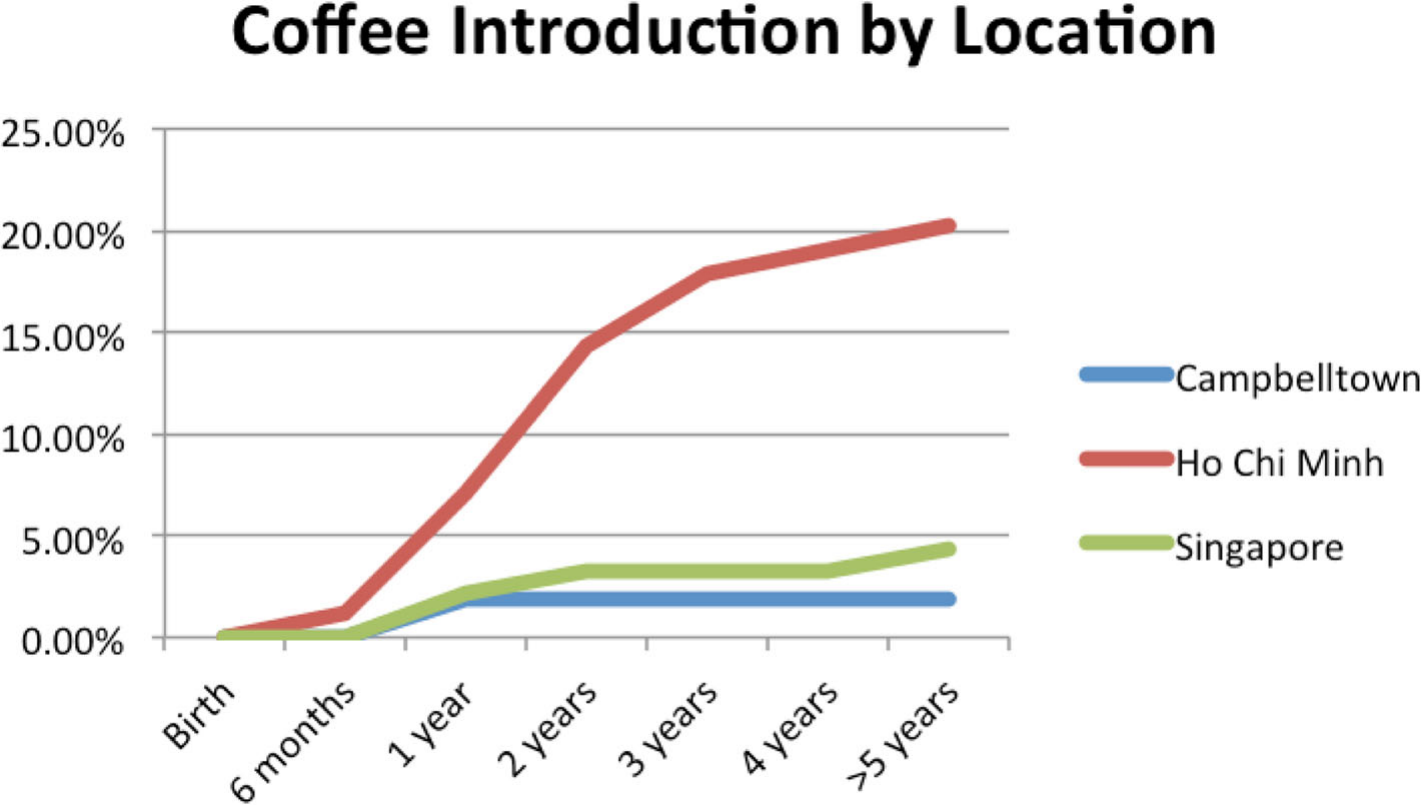
Number of participants who introduced coffee by age across locations

**Fig. 12:**
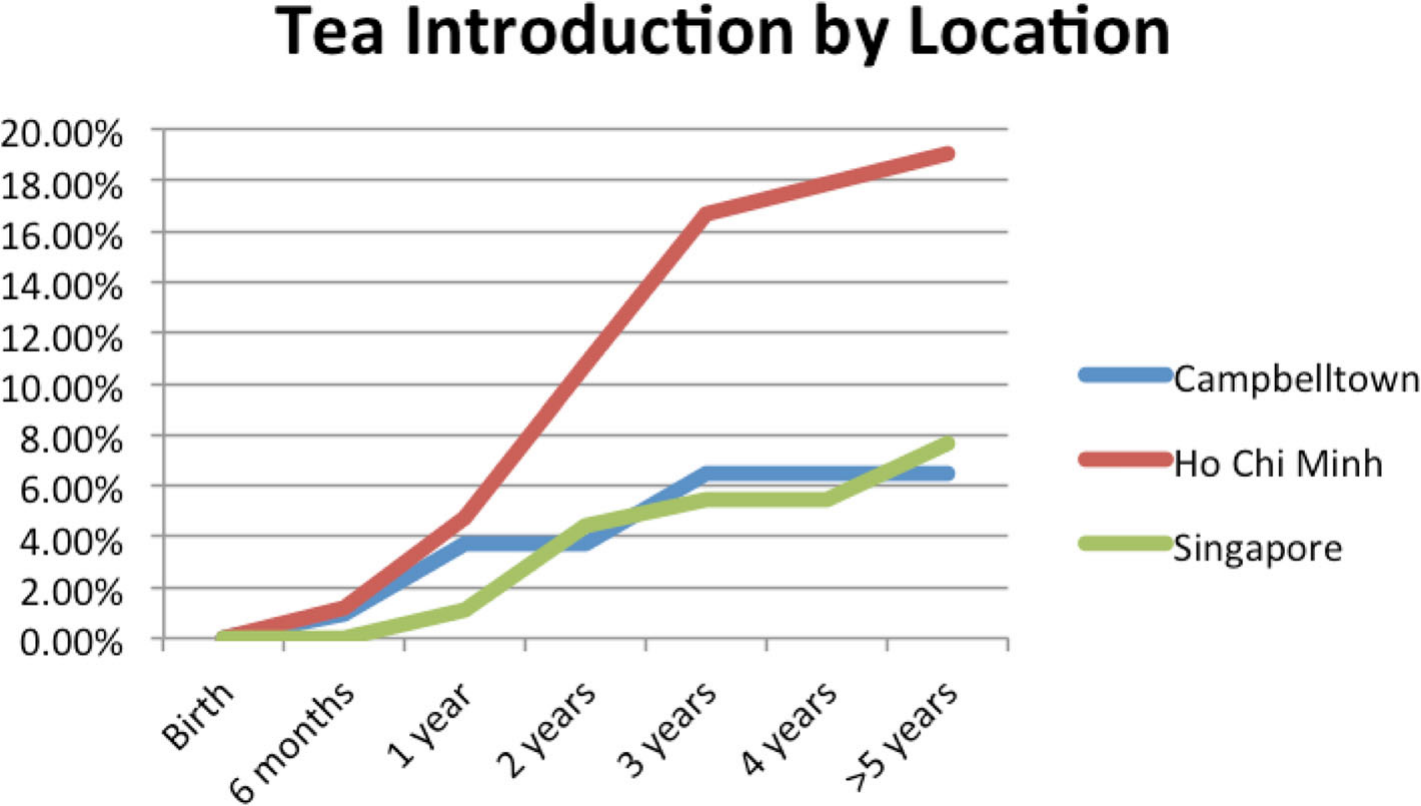
Number of participants who introduced tea by age across locations

**Fig. 13:**
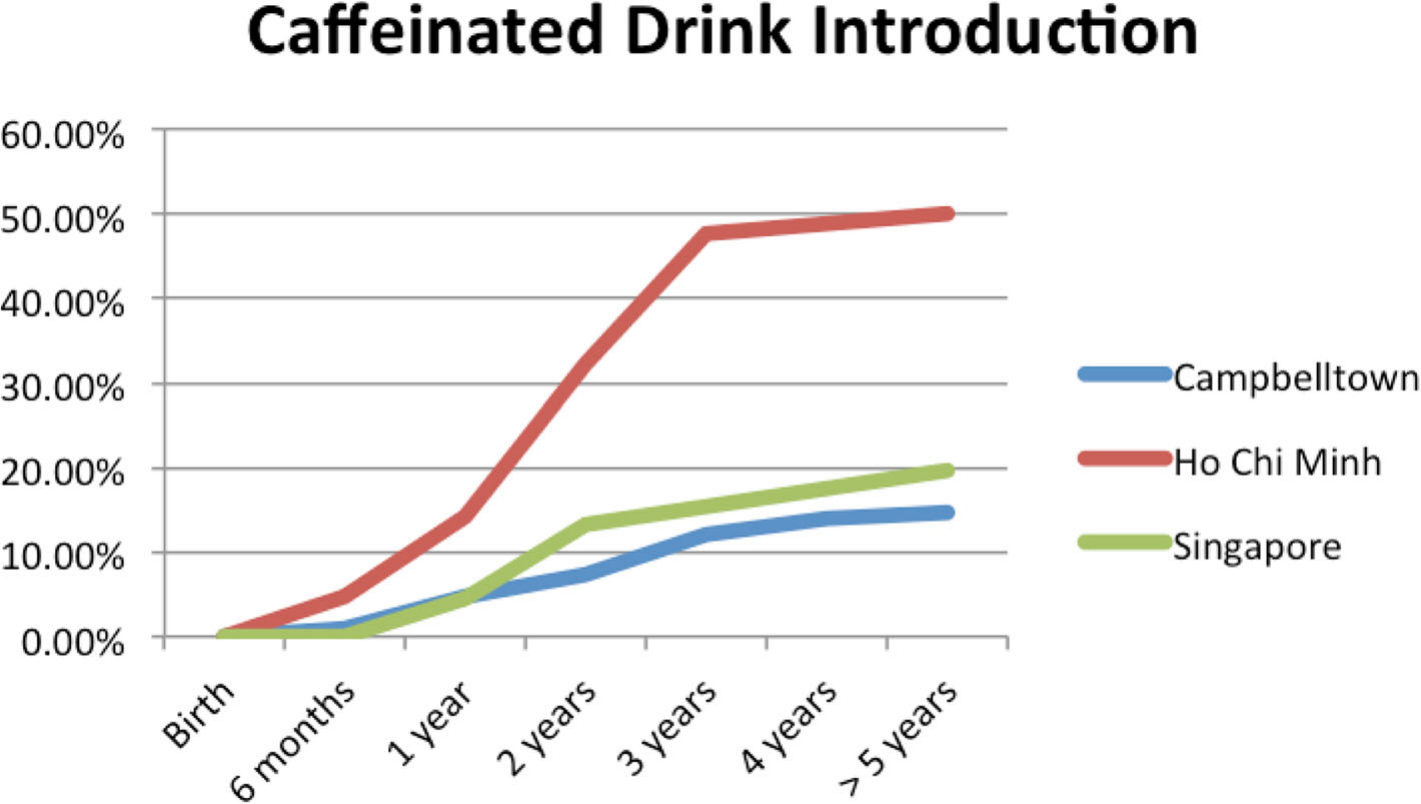
Number of participants who introduced any caffeinated drink by age across locations

Caffeinated soft drinks (CDs), were introduced at an early age in all localities (Figs. 9, 10, 11, 12 and 13) In HCMC and Campbelltown they were introduced by six months to 4.8% and 0.9% of children respectively. By one year of age, they had been introduced to14.3% of children in HCMC, 4.6% in Campbelltown and 4.4% in Singapore. By three years of age, they had been introduced to 47.6% of children in HCMC, 12.0% in Campbelltown and 15.4% in Singapore. These differences were statistically significant for both age and rate of introductions *p* < 0.05*.* HCMC had introduced more CDs and at an earlier age than the other sites at a statisically significant rate to children (≤3 years old).

Table 1 shows the percentages of various drinks introduced to children (less than six years) in the three sites revealing high rates of consumption of drinks with elevated calorie and caffeine content in all areas. The rates were significantly increased in Vietnam for all types of drinks except 100% fruit juice.

**Table 1:** Rates of introduction of various drinks (ages 0–6 years old) according to location

	Ho Chi Minh City, *n* = 84 (%)	Campbelltown, *n* = 108 (%)	Singapore, *n* = 91 (%)	Total, *n* = 283 (%)
Water	83 (98.8%)	96 (88.%)	77 (84.6%)	256 (90.5%)
Cow’s milk	23 (27.4%)	70 (64.8%)	28 (31.1%)	125 (44.2%)
Cordial	12 (14.2%)	37 (34.3%)	14 (15.4%)	63 (22.2%)
Flavoured milk	36 (42.9%)	43 (39.8%)	23 (25.3%)	102 (36.0%)
100% Fruit juice	43 (51.2%)	55 (50.9%)	36 (29.6%)	134 (47.3%)
Fruit Drink	61 (72.6%)	44 (40.7%)	24 (26.4%)	129 (45.6%)
Non-caffeinated soft-drink	38 (45.2%)	22 (20.4%)	15 (16.5%)	75 (26.5%)
Caffeinated soft-drink	32 (38.1%)	10 (9.3%)	11 (12.1%)	53 (18.7%)
Energy drinks	10 (12.0%)	1 (0.9%)	0	11 (3.9%)
Coffee	17 (20.2%)	2 (1.9%)	4 (4.4%)	23 (8.1%)
Tea	16 (19.0%)	7 (6.5%)	7 (7.7%)	30 (10.6%)

Flavoured milk (Fig. 5) was introduced by the age of six months by participants at 4.8% in HCMC, 0.9% in Campbelltown, and 1.1% in Singapore. At one year, 22.6% of participants in HCMC had been introduced flavoured milk, compared to 7.4% in Campbelltown and 5.5% in Singapore. By three years of age, 41.7% of participants in HCMC had received flavoured milk, compared to 36.1% in Campbelltown and 24.2% in Singapore (Table 1). These differences were statistically significant by rate of introductions *p* < 0.05, but not by age of introduction. HCMC had introduced more flavoured milk than the other sites at a statistically significant rate to children (≤3 years old) but there was no significant difference between the sites at the age of initial introduction.

100% fruit juice (Fig. 6) was introduced by 13.1% of participants in HCMC by the age of six months, compared with 7.4% in Campbelltown and 8.8% in Singapore. At one year, 44.1% of participants in HCMC had been introduced 100% fruit juice, compared to 21.3% in Campbelltown and 26.4% in Singapore. By three years of age, 50% of participants in Campbelltown and HCMC had been introduced 100% fruit juice, compared to 38.5% in Singapore (Table 1). These differences were not statistically significant by rate of introductions but by age of introduction *p* < 0.05*.* HCMC had introduced 100% fruit juice at an earlier age than the other sites at a statistically significant rate to children (≤3 years old) but there was no significant difference between the sites for the number of introductions.

Fruit drink (Fig. 7) was introduced by 22.6% of participants in HCMC by six months, compared with 6.5% in Campbelltown and 4.4% in Singapore. By one year, 59.5% of participants in HCMC had been introduced fruit drinks, compared to 20.4% in Campbelltown and 15.4% in Singapore. By three years, the rate rose to 72.6% of in HCMC, 40.7% in Campbelltown and 26.4% in Singapore (Table 1). These differences were statistically significant by both rate and age of introductions *p* < 0.05*.*

Non-caffeinated soft-drinks (Fig. 8) were introduced by 2.4% of participants in HCMC by six months, but none were introduced in Campbelltown and Singapore. At one year, the rate rose to 11.9% of participants in HCMC, compared to 1.9% in Campbelltown and 5.5% in Singapore. By three years, 44.1% of participants in HCMC had been introduced non-caffeinated soft-drinks, compared to 18.5% in Campbelltown and 12.1% in Singapore (Table 1). The differences between the locations were statistically significant by the rate of introductions *p* < 0.05, but not by the age of introduction.

Caffeinated soft-drinks (Fig. 9) were introduced by six months by 3.6% of participants in HCMC, but by none in Campbelltown and Singapore. By one year, 9.5% of participants in HCMC had introduced caffeinated soft drinks, compared to 0.9% in Campbelltown, and 1.1% in Singapore. By three years, the rate rose to 36.9% in HCMC, 7.4% in Campbelltown and 8.8% in Singapore (Table 1). The differences between the locations were statistically significant by the rate of introductions *p* < 0.05, but not by the age of introduction.

HCMC had the highest percentage of participants who had received CDs at an early age: (Fig. 11) by six months, 1.2%; by one year 2.4%, and by three years 10.7% (Table 1).

Coffee (Fig. 11) was introduced by 1.2% of participants in HCMC by six months, but by none in Campbelltown and Singapore. At one year, the rate had risen to 7.1%of participants in HCMC, 1.9% in Campbelltown, and 2.2% in Singapore. By three years, the rate had risen to 17.9% in HCM and to 3.3% in Singapore but was stable at 1.9% in Campbelltown (Table 1). These differences were statistically significant by the rate of introductions *p* < 0.05*,* but not by the age of introduction.

Tea (Fig. 12) was introduced by six months in 4.8% of participants in HCMC, 0.9% in Campbelltown, and 1.1% in Singapore. By one year the rate had risen to 10.7% of participants in HCMC, 3.7% in Campbelltown, and 4.4% in Singapore. By three years, it had risen to 16.7% in HCMC, 6.5% in Campbelltown and 5.5% in Singapore (Table 1). These differences were statistically significant by rate of introductions *p* < 0.05*,* but not by the age of introduction*.*

A significant association between the age of introduction of the following drinks (Table 2): water (*p* < 0.005), cordial (*p* < 0.005), 100% fruit juice (*p* < 0.05), and fruit drink (*p* < 0.005). Post-hoc Tukey HSD analyses showed that HCMC introduced water earlier than Campbelltown (*p* < 0.01), and Singapore (*p* < 0.00). HCMC also introduced cordial earlier than both Campbelltown (*p* < 0.00), and Singapore (*p* < 0.00). 100% fruit juice was introduced earlier in HCMC compared to Campbelltown (*p* < 0.04) and fruit drink was introduced earlier in HCMC compared to Campbelltown (*p* < 0.00). There was no statistical difference in the time of introduction of infant formula, cow’s milk, flavoured milk, non-caffeinated soft drinks, caffeinated soft drinks, coffee, tea and solid foods between the different locations. Infant formula, non-caffeinated soft drinks, and caffeinated soft drinks were being introduced within months of birth (by 6 months). Only in Vietnam were high energy drinks being introduced to children by 6 years of age. 9 respondents introduced energy drinks from HCMC but none from the other locations.

**Table 2:** One-way analysis of variance (ANOVA) between locations for the age of introduction of drinks

	Sum of squares	df	Mean square	F	*p*
Water	3.562	2	1.781	7.321	0.001
Cow’s milk	4.503	2	2.252	2.549	0.082
Cordial	37.843	2	18.921	18.339	0.000
Flavoured milk	4.501	2	2.250	1.774	0.175
100% Fruit juice	8.566	2	4.283	3.120	0.047
Fruit Drink	12.635	2	6.317	6.563	0.002
Non-caffeinated soft-drink	0.843	2	0.422	0.326	0.723
Caffeinated soft-drink	3.983	2	1.991	1.411	0.253
Coffee	1.214	2	0.607	0.269	0.767
Tea	5.181	2	2.590	1.200	0.317

Significant differences in parent education levels were demonstrated F(2280) = 15.40, *p* < 0.00). Tertiary education was completed by 46.4% (*n* = 39) in HCMC compared to 75.0% (*n* = 81) in Campbelltown and 75.8% (*n* = 69) in Singapore. 36.9% (*n* = 31) of respondents in HCMC did not complete secondary school.

## Discussion

The study revealed differences in feeding patterns between the sites. First, though breast feeding rates were lower in all areas than recommended by WHO (24.72%), the rates were significantly lower in Vietnam (14.47%). Second, overall rates of introduction to high caloric beverages were high, but particularly in Vietnam. Third, overall rates of introduction of caffeinated drinks were high (Avg: 17.58% by two years of age) especially in HCMC.

Vietnam’s ‘Alive and Thrive’ organisation seeks to increase breast feeding in that country and suggests several reasons for its low rate. First, is the popular misconception that Vietnamese women produce insufficient quantity and quality of breast milk. Second, is the practice of giving water after breastfeeding to clean the child’s mouth and reduce lingering thirst, with the subsequent effect of reduced stimulation for milk production. Third, is the short official maternity leave of less than 4 months. Fourth, the aggressive marketing of infant formula. Fifth, the paucity of adequately trained breast feeding educators [48]. Lower levels of education are associated with lower rates of breast feeding and this association was confirmed in our study.

Efforts were introduced in 2010 to counter these obstacles to breast feeding and included extension of maternity leave to 6 months, banning advertising of alternatives to breastmilk, and introduction of lactation support programs in 70 locations, but rates have shown little improvement [49, 50].

HCMC’s lower compliance with infant feeding guidelines is consistent with the lower rate of public health spending in Vietnam which, per year, is only $3.45 per capita, compared to Australia’s $41.70 and Singapore’s $56.90 [46, 47, 51, 52]. This low rate of spending on health promotion in Vietnam contrasts with high rates of investment by infant formula companies. From 2010 to 2013, an estimated USD $13 million was spent on advertising on infant formulas to secure a revenue of $1.23 billion [53, 54].

Supplementation of breast feeding with infant formula was initiated earlier in Campbelltown where 37% of respondents declared supplementation had been introduced at birth, compared with 26% in HCMC and 24% in Singapore. As infant formula is reported to be associated with development of obesity, this early introduction in Campbelltown may contribute to the higher rate of childhood obesity in that site (25% compared to 16.5 in HCMC and 11% in Singapore) [34–39, 55].

High calorie drinks were introduced earlier in Vietnam than in the other sites, coinciding with investment by their manufacturers. The most popular STING energy drink company is reported to have invested US$250 million from 2010 to 2013 [56], and Coca Cola announced a new US$300 million investment in 2012, increasing its total to US $500 million in the years 2010–2015 [57]. These companies are reported to have generated revenue of US$56 million and US$113 million respectively in 2010 [56, 57].

High calorie drinks are promoted as being healthy but provide little else than carbohydrate. Merely one serving provides 480-675 kJ [58, 59], or 15% of the recommended daily energy requirements, thus contributing to obesity [22, 60–62]. Also the volume consumed competes with consumption of proteins, vitamins and minerals [18, 22]. Parents may be misled by false advertising that these juices are ‘healthy’ and need government protection [63].

Caffeine is a psychoactive stimulant, reported to contribute to hyperactivity [21, 64], addictive behaviours [30], and depression [65]. According to Goldman [21] caffeinated energy drinks are not recommended to children, due to its potential harmful effects. While Temple [30] and Beckford [66] highlight rising concerns with the introduction of caffeinated beverages to children and the need for further research into toxicity. Caffeine was introduced in all sites, but earlier and to many more children in Vietnam, in coffee, energy drinks and tea.

Our study has shown differences in rates of breast feeding between the sites, and that the differences correlate with introduction and consumption of high calorie feeds, all of which are known to correlate with obesity [5, 16, 67]. While rates of obesity have increased in recent years in all sites, the rates are higher in Vietnam in association with reduced breast feeding and increased caloric consumption in infancy [55, 68–70]. In Vietnam, overall, obesity rates increased in children, from 3.2% to 6.3% between 2002 and 2005 [55, 71]. In HCMC, in 2010, the rate of overweight/obese adolescents was 21% [55]. However, in Vietnam we note that urban regions experience a much greater increase in rates of obesity as compared to the suburban/rural areas [38, 55, 72, 73]. In Australia, the rate of overweight children has increased from 21% in 1995 to 25.7% in 2011–12 [37, 68]. In Singapore, child obesity rates remained steady at 12% between 2010 and 2013 [74].

Limitations to the study included its limited sample size due to allocated time frame of data collection, the possible feeling of intimidation of respondents by a supervised questionnaire in cultures not familiar with total freedom of expression, and the lack of direct measurement of obesity rates amongst the child participants. The fact the questionnaires were distributed near government health facilities may have increased this intimidation as well as selection bias.

### Future research

HCMC’s poorer compliance with WHO infant feeding recommendations is of concern. Continuing research on factors that influence infant feeding practices is basic to the development of appropriate interventions in all three sites to reduce subsequent rates of obesity and its complications.

## Conclusions

There was statistical significance between the duration of exclusive breastfeeding, the age of introduction of cordial, flavoured milk and fruit drink between the regions, as well as the educational level of respondents. Vietnam had lower rates of breast feeding but higher rates of introduction of HCBs and CDs, which were associated with lower rates of education achievement. These feeding practices are reflected in rising obesity rates and the need for greater programs of education and perhaps government interventions to favour breast feeding and restrict false advertising of HCBs and CDs.

## Additional files

Additional file 1: The infant feeding survey. (DOCX 51 kb)

Additional file 2: Descriptive statistics of the carer/parent. (DOCX 17 kb)

Additional file 3: Descriptive statistics of the child. (DOCX 16 kb)

## Abbreviations

CB: Caffeinated beverages
HCB: High calorie beverages
HCMC: Ho Chi Minh City, Vietnam
NGO: Non-governmental organisation
SWS: Campbelltown (South Western Sydney), New South Wales, Australia
USD: United States of America dollar
