# Supplementary material for: Exploring infant feeding practices: cross-sectional surveys of South Western Sydney, Singapore, and Ho Chi Minh City
Source: BMC Pediatr. 2017 Jun 13;17:145. doi: 10.1186/s12887-017-0902-0 (PMC5470214; doi:10.1186/s12887-017-0902-0)
Supplement: Supplementary file 1 — The infant feeding survey. (DOCX 51 kb) [file 12887_2017_902_MOESM1_ESM.docx]

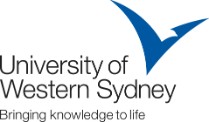


**Infant Feeding Survey**

By completing this survey, you acknowledge that you have read and understood the participant information sheet, and you consent to participate in this research project.

**Introductory/Demographic Questions**

1. Please circle your gender Male Female

2. Please state your age:

3. Please circle which option corresponds to your highest level of completed education:

a. No schooling

b. Some primary school

c. Completed primary school

d. Some secondary (high) school

e. Completed secondary school (Yr 12)

f. TAFE qualification

g. University degree

h. Other (please specify)

4. Is English the language spoken most of the time at home? Yes No

5. Were you born in Australia? Yes No

6. What is the child’s cultural background?

a. Indigenous Australian

b. Pacific Island

c. Caucasian

d. Asian

e. Subcontinent

f. Middle East

g. Africa

h. Other (please specify)

7. How many children do you care for? (please circle)

1. One

b. Two

c. Three

d. Four or more

**Questions about your child’s diet**

Answer all of the following questions for your YOUNGEST child only

8. Please state the age of your YOUNGEST child:

9. Circle the gender of your YOUNGEST child: Male Female

10. Was your child ever breastfed? Yes No Don’t Know (*If No or Don’t Know go to question 13)*

*In the following questions the phrase ‘EXCLUSIVELY breastfed’ is used. ‘Exclusively breastfed’ means that the infant/child received only breast milk and no other liquids or solids, excluding medicine.*

11. Was your child ever EXCLUSIVELY breastfed? Yes No Don’t Know (*If No or Don’t Know go to question 13)*

12. Until what age was your child EXCLUSIVELY breastfed?

a. 0-1 month

b. 1-2 months

c. 2-3 months

d. 3-4 months

e. 4-5 months

f. 5-6 months

g. More than 6 months

h. My child is still exclusively breastfed

i. Don’t know

13. Please complete the following table regarding infant feeding:

| **Food** | **Have you introduced this drink into your child’s diet?** | **Approximately how old was your child when you introduced this drink?** | **How often did your child consume this drink?** |
| --- | --- | --- | --- |
| Infant formula (breast milk substitute) | No  Yes  Don’t know | ___ yrs ___ months  NA | More than once a day  Once daily  Two to three times per week  Weekly  Monthly  Less than once a month |
| Water | No  Yes  Don’t know | ___ yrs ___ months  NA | More than once a day  Once daily  Two to three times per week  Weekly  Monthly  Less than once a month |
| Cow’s milk | No  Yes  Don’t know | ___ yrs ___ months  NA | More than once a day  Once daily  Two to three times per week  Weekly  Monthly  Less than once a month |
| Cordial | No  Yes  Don’t know | ___ yrs ___ months  NA | More than once a day  Once daily  Two to three times per week  Weekly  Monthly  Less than once a month |
| Flavoured milk (e.g. chocolate milk) | No  Yes  Don’t know | ___ yrs ___ months  NA | More than once a day  Once daily  Two to three times per week  Weekly  Monthly  Less than once a month |
| 100% Fruit juice | No  Yes  Don’t know | ___ yrs ___ months  NA | More than once a day  Once daily  Two to three times per week  Weekly  Monthly  Less than once a month |
| Fruit drink (sweetened, diluted fruit juice) | No  Yes  Don’t know | ___ yrs ___ months  NA | More than once a day  Once daily  Two to three times per week  Weekly  Monthly  Less than once a month |
| Non-caffeinated soft-drinks (e.g. lemonade) | No  Yes  Don’t know | ___ yrs ___ months  NA | More than once a day  Once daily  Two to three times per week  Weekly  Monthly  Less than once a month |
| Caffeinated soft-drinks (e,g cola) | No  Yes  Don’t know | ___ yrs ___ months  NA | More than once a day  Once Daily  Two to three times per week Weekly  Monthly  Less than once a month |
| “Energy” drinks (such as those with high caffeine, guaran, taurine or ginseng) | No  Yes  Don’t know | ___ yrs ___ months  NA | More than once a day  Once Daily  Two to three times per week  Weekly  Monthly  Less than once a month |
| Coffee | No  Yes  Don’t know | ___ yrs ___ months  NA | More than once a day  Once Daily  Two to three times per week  Weekly  Monthly  Less than once a month |
| Tea | No  Yes  Don’t know | ___ yrs ___ months  NA | More than once a day  Once Daily  Two to three times per week  Weekly  Monthly  Less than once a month |
| Solid Foods (ingredients)  Specify ______ | No  Yes  Don’t know | ___ yrs ___ months  NA | More than once a day  Once Daily  Two to three times per week  Weekly  Monthly  Less than once a month |
