# Supplementary material for: Exploring infant feeding practices: cross-sectional surveys of South Western Sydney, Singapore, and Ho Chi Minh City
Source: BMC Pediatr. 2017 Jun 13;17:145. doi: 10.1186/s12887-017-0902-0 (PMC5470214; doi:10.1186/s12887-017-0902-0)
Supplement: Supplementary file 2 — Descriptive statistics of the carer/parent. (DOCX 17 kb) [file 12887_2017_902_MOESM2_ESM.docx]

**Table: Descriptive statistics of the carer/parent**.

|  | | Number (N) | % of total population |
| --- | --- | --- | --- |
| Gender | Male | 37 | 12.9 |
|  | Female | 246 | 85.7 |
| Age Groups | 20-24 | 17 | 6.0 |
|  | 25-29 | 62 | 21.9 |
|  | 30-34 | 89 | 31.4 |
|  | 35-39 | 75 | 26.5 |
|  | 40-44 | 21 | 7.4 |
|  | 45-49 | 6 | 2.1 |
|  | ≥ 50 | 11 | 3.9 |
| Education level | No Schooling | 0 | 0 |
|  | Some Primary School | 2 | 0.7 |
|  | Completed Primary School | 6 | 2.1 |
|  | Some Secondary School | 49 | 17.1 |
|  | Completed Secondary School | 37 | 12.9 |
|  | TAFE Qualification or equivalent | 58 | 20.2 |
|  | University Degree | 112 | 39.0 |
|  | Other (e.g. postgraduate studies) | 19 | 6.6 |
| English most spoken language | Yes | 134 | 46.7 |
|  | No | 145 | 50.5 |
| Cultural Background | Aboriginal/Indigenous Australian | 8 | 2.8 |
|  | Torres strait and Pacific Islands | 9 | 3.1 |
|  | Caucasian | 50 | 17.4 |
|  | Asian | 142 | 49.5 |
|  | Subcontinent | 22 | 7.7 |
|  | Middle East | 4 | 1.4 |
|  | African | 4 | 1.4 |
|  | Other (e.g. Malay, Indonesian, Filipino, and mediterranian) | 42 | 14.6 |
| Number of Children | 1 | 111 | 38.7 |
|  | 2 | 109 | 38.0 |
|  | 3 | 35 | 12.2 |
|  | 4 or more | 28 | 9.8 |
