# Supplementary material for: Exploring infant feeding practices: cross-sectional surveys of South Western Sydney, Singapore, and Ho Chi Minh City
Source: BMC Pediatr. 2017 Jun 13;17:145. doi: 10.1186/s12887-017-0902-0 (PMC5470214; doi:10.1186/s12887-017-0902-0)
Supplement: Supplementary file 3 — Descriptive statistics of the child. (DOCX 16 kb) [file 12887_2017_902_MOESM3_ESM.docx]

**Table:** **Descriptive statistics of the child.**

|  | | Count (N) | Count (%) |
| --- | --- | --- | --- |
| Age of youngest child | 0 - 11 | 78 | 28 |
|  | 12 – 23 | 71 | 25.4 |
|  | 24 – 35 | 41 | 14.7 |
|  | 36 – 47 | 48 | 17.2 |
|  | 48 – 59 | 20 | 7.2 |
|  | 60 - 72 | 21 | 7.5 |
| Gender of youngest child | Male | 150 | 53.0 |
|  | Female | 133 | 47.0 |
| Youngest child breastfed | Yes | 237 | 83.7 |
|  | No | 46 | 16.3 |
|  | Don’t Know | 0 | 0 |
| Youngest child exclusively breastfed | Yes | 166 | 60.6 |
|  | No | 108 | 39.4 |
|  | Don’t Know | 0 | 0 |
| Period of exclusively breastfed | 0-1 months | 36 | 18.7 |
|  | 1-2 months | 10 | 5.2 |
|  | 2-3 months | 14 | 7.3 |
|  | 3-4 months | 17 | 8.9 |
|  | 4-5 months | 14 | 7.3 |
|  | 5-6 months | 33 | 17.2 |
|  | more than 6 months | 36 | 18.8 |
|  | my child is still exclusively breastfed | 31 | 16.1 |
|  | Don’t Know | 1 | 0.5 |
|  | Total | 192 | 100.0 |
